# Magnetic resonance imaging and next-generation sequencing for the diagnosis of cystic echinococcosis in the intradural spine: a case report

**DOI:** 10.1186/s13256-023-04197-1

**Published:** 2023-11-10

**Authors:** Xiaojie Lao, Diefei Hu, Lei Ji, Tingzheng Zhan, Tiantian Li, Shuming Luo, Xianli Xu, Chunlan Zhang, Maowei Chen

**Affiliations:** 1 Beijing Ditan Hospital, Capital Medical University, Beijing, 100015 China; 2 Department of Infectious Diseases, Guangxi Medical University First Affiliated Hospital, Nanning, China; 3 Department of Infectious Diseases, Wuming Hospital of Guangxi Medical University, Nanning, China; 4 Department of Parasitology, Guangxi Medical University, Nanning, Guangxi China

**Keywords:** Next-generation sequencing, Cystic echinococcosis, Hydatid disease, Case report

## Abstract

**Background:**

Cystic echinococcosis (CE) is a parasitic zoonotic disease caused by the larval stage of *Echinococcus granulosus*. The liver and lungs are the most common sites for infection. Infection of the intradural spine is rare.

**Case presentation:**

A 45-year-old woman of Han ethnicity presented with a chronic history of recurrent lumbar pain. Magnetic resonance imaging of the lumbar spine revealed the classical characteristic of multiple cystic lesions of variable sizes, manifesting a “bunch of grapes” appearance, localized within the spinal canal at the L4–L5 vertebral level. In the meanwhile, metagenomic next-generation sequencing identified *Echinococcosis granulosa*. The patient underwent surgery to remove the cyst entirely and subsequently took albendazole 400 mg orally twice daily for 6 months.

**Conclusion:**

Spinal CE should be suspected in patients with multiple spinal cystic lesions and zoonotic exposure. metagenomic next-generation sequencing serves as a robust diagnostic tool for atypical pathogens, particularly when conventional tests are inconclusive. Prompt and aggressive treatment for spinal cystic echinococcosis is imperative, and further research is warranted for improved diagnostic and therapeutic strategies.

## Background

Cystic echinococcosis (CE) is a globally distributed parasitic zoonosis in humans caused by the larval stage of the tapeworm *Echinococcus granulosus* (*E. granulosus*). The disease is a significant public health concern and endemic in various regions, particularly affecting pastoral and agricultural settings where close contact between humans and livestock is common [1]. While the liver and lungs are the most commonly affected organs, accounting for approximately 90% of all cases, spinal involvement is a rare manifestation of the disease. The scarcity of spinal CE cases makes diagnosis and treatment particularly challenging. In this case, we report a rare case of spinal cystic echinococcosis.

## Case presentation

A 45-year-old woman of Han ethnicity presented to our emergency department with complaints of a 4-year history of recurrent lumbar pain and weakness in the right lower limb. The pain was related to the activity and relieved on lying down. She had no fever, night sweats, and weight loss. She was an agricultural worker with occupational exposure to wooded environments and had a documented history of canine interaction. In her past medical history, we noticed that she initially underwent a discectomy at the L4–L5 level 4 years prior, owing to persistent low back pain that was unresponsive to conservative measures. However, despite initial relief, she experienced symptom recurrence 2 years postoperatively. Subsequent imaging revealed a cystic lesion at the same spinal level, necessitating a second surgical intervention. Histopathological analysis of the resected tissue indicated chronic granulomatous inflammation but was inconclusive for specific pathogens. Regrettably, the patient’s lumbar pain recurred 8 months following the second procedure, prompting further diagnostic scrutiny.

The patient’s lower lumbar and sacral area was tender. She had reduced sensation in her left lower limbs at L5–S1 dermatomes. The straight leg raising test on the left leg was limited to 30°, and the right leg was 45°. Power grade and tendon reflexes in both lower limbs were normal. The complete blood count test and the C-reactive protein level were normal. Magnetic resonance imaging (MRI) was performed.

**Fig. 1:**
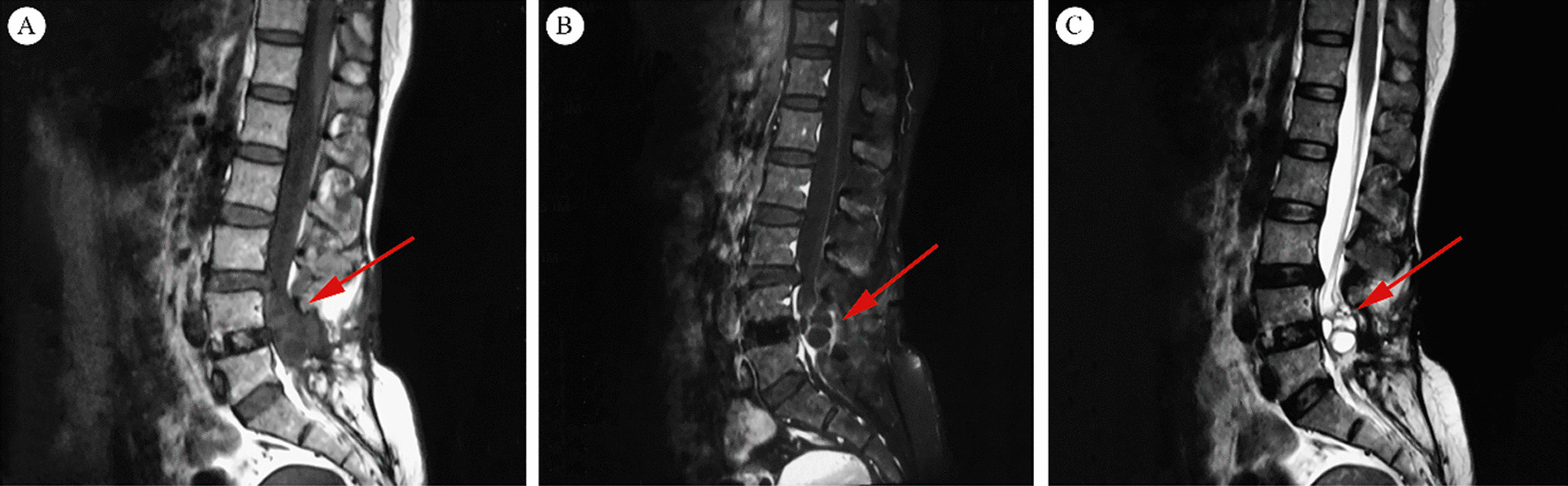
Magnetic resonance images of the lumbar spine. **A** Sagittal T1-weighted magnetic resonance imaging showing multiple low signal intensity lesions (red arrow). **B** Sagittal T1-weighted magnetic resonance imaging with intravenous gadolinium contrast medium showing the same lesion with low signal intensity, but the edge is enhanced (red arrow). **C** Sagittal T2-weighted MRI showing multiple high signal lesions (red arrow)

MRI showed that there were multiple cystic lesions of variable sizes in the spinal canal at the L4–L5 level, which appeared as a “bunch of grapes” [2] (Fig. 1). The cyst wall was enhanced in T1-weighted MRI with contrast medium and consistent low signal intensity in T2-weighted images. Cyst fluids showed water-like signals with low signal intensity in T1-weighted images and high signal intensity in T2-weighted images.

Such radiographic findings prompted us to perform metagenomic next-generation sequencing (mNGS) on the previous tissue slices of surgery. The result confirmed the infection of *Echinococcus granulosus* (*E. granulosus*).

The patient underwent a PET/CT scan of the whole body to rule out potential CE cysts in another possible site of involvement [3]. No other extraspinal hydatid cysts were found. The patient underwent surgery to excise the cysts entirely and started walking again within 3 weeks. Subsequently, the patient was prescribed albendazole (400 mg orally twice daily) for 6 months. No recurrence was observed at 1-year follow-up after discharge.

## Discussions

Our case report elucidates the diagnostic and therapeutic challenges associated with spinal CE, a rare but severe manifestation of *Echinococcus granulosus* infection. The patient’s occupational exposure to wooded areas and documented interaction with canines heightened the index of suspicion for a zoonotic etiology. Her recurrent lumbar pain, despite two prior surgical interventions, necessitated a more comprehensive diagnostic approach.

Often missed in clinical evaluations, CE, also known as hydatid diseases (HD), is a neglected parasitic zoonotic disease caused by the larval stage of *E. granulosus* [4] and found in canines (definitive host), sheep, cattle, goats, and pigs (intermediate hosts). CE is mainly endemic in agricultural and animal husbandry countries and rarely reported in other regions. Because the hydatid cyst grows slowly for several years, most patients remain asymptomatic during the initial phases of infection, thereby missing the optimal treatment stage. CE manifests as progressively enlarging space-occupying lesions in the liver, lungs, and other organs. The rupture of a cyst can lead to the spread of new cysts, inducing anaphylactic shock and potentially causing death [5]. The overall mortality rate of CE is around 2%, with an obvious increase in untreated or inadequately treated patients [6].

In previous studies, cystic lesions can be resided in any organ of the human body through blood. The liver (91.9%) [7, 8] is the most frequent site for cystic lesions in hydatid disease, followed by the lung and brain [9, 10]. Infection involving the spine is rare and only accounts for 0.2–1% of all cases [11].

The clinical manifestations of intradural spinal CE depended on the cyst’s location and size. It usually manifests as compression symptoms that cause back pain and neurological disability, which is also similar to other space-occupying lesions in the spinal canal and easily missed or misdiagnosed. Differential diagnoses of lesions located at the intradural spinal cord included schwannoma, metastasis, abscess, spinal tuberculosis, and parasites.

Traditional laboratory tests for echinococcosis include serological tests, X-rays, and MRIs. While serological tests offer some diagnostic utility, they are not invariably reliable. Radiographic techniques, including X-ray and MRI, can show the lesion site of most organs but are incapable of pathogen identification. In contrast, as a hypothesis-free, high-throughput sequencing technique, mNGS could detect a broad spectrum of nucleic acids in multiple specimens, such as blood, pleural fluid, cerebrospinal fluid, and tissue specimens [12]. All pathogens in specimens, including viruses, bacteria, fungi, and parasites, can be detected without bias based on sequence information by comparing with established microbial sequence databases. Despite limitations such as high cost and the absence of standard interpretation of results, our case illustrates the invaluable role of mNGS in diagnosing unexplained, rare, and emerging infectious diseases when traditional laboratory tests fail [13].

The treatment of CE is based on surgical resection and drug treatment [14]. Surgery is the first choice for spinal CE, which should excise the CE cysts in full without rupture. Otherwise, cyst fluid can result in an anaphylactic reaction and a recurrence [14]. Albendazole (15 mg/kg/day) could help reduce the recurrence risk following surgery [15, 16]. In addition, regular follow-up with MRI is critical for patients. In this case, the patient recovered well and there was no recurrence after surgery and medication.

In summary, this case underscores the importance of considering spinal CE in the differential diagnosis of recurrent lumbar pain, especially in patients with relevant occupational or environmental exposures. Advanced diagnostic methods such as mNGS can be invaluable in complex cases, guiding clinicians toward appropriate and effective treatment strategies.

## Conclusion

1. In patients presenting with multiple spinal cystic lesions, spinal cystic echinococcosis should be considered, especially in patients with documented zoonotic exposure to canines or ovines.
2. This case showed that mNGS could be a powerful tool for identifying uncommon pathogens in clinical specimens when results from routine tests are negative and the patient’s condition is undiagnosed. mNGS offers an innovative strategy for diagnosing neurological infections with nonspecific symptoms, thereby facilitating timely clinical decision-making.
3. Spinal CE is an uncommon but serious manifestation of cystic echinococcosis that requires prompt diagnosis and aggressive treatment. Further research is needed to understand the epidemiology and pathogenesis of spinal CE, as well as to develop more effective diagnostic and therapeutic strategies.

## Data Availability

Data sharing is not applicable to this article as no datasets were generated or analyzed during the current study.

## Abbreviations

mNGS: Metagenomic next-generation sequencing
MRI: Magnetic resonance imaging
CE: Cystic echinococcosis
HD: Hydatid diseases
